# Supplementary material for: Validity and reliability of a teledentistry survey among dental practitioners in Saudi Arabia
Source: PeerJ. 2024 Jan 31;12:e16834. doi: 10.7717/peerj.16834 (PMC10838065; doi:10.7717/peerj.16834)
Supplement: Supplemental Information 3 [file peerj-12-16834-s003.pdf]

QUESTIONNAIRE

1. **Nationality:**
   a. Saudi
   b. Non-Saudi
2. **Age:**
   a. 23–34 yr
   b. 35–44 yr
   c. 45–54 yr
   d. 55–64 yr
   e. > 65 yr
3. **Gender:**
   a. Male
   b. Female
4. **Qualification**
   a. General Dental Practitioner
   b. Dental Specialist
   c. Dental consultant
5. **Years of work experience in dentistry:**
   a. 1-5 yr
   b. 6-10 yr
   c. 11-15 yr
   d. > 16 yr
6. **Work setting of main job:**
   a. Private
   b. Public
   c. Both
7. **The average general use of internet (in hours/day) - use of Internet for purposes other than work:**
   a. < 1 Hour
   b. 2-4 Hour
   c. 5-7 Hour
   d. 8-10 Hour
   e. >10 Hour
8. **The average work-related use of Internet (in hours/day) - Use of Internet for work purposes only:**
   a. < 1 Hour
   b. 2-4 Hour
   c. 5-7 Hour
   d. 8-10 Hour
   e. >10 Hour
9. **How do you most commonly communicate with colleagues (e.g., other dentists or Specialists) - You can check more than one box if you wish.**
   a. In person
   b. Phone
   c. E-mail
   d. Social media
   e. Videoconferencing
   f. Forum

10. **On a scale of 1 (Strongly disagree) to 5 (Strongly agree) how would you agree with the following statements regarding dentists' perceptions of usefulness of teledentistry system:**
- Teledentistry would provide adequate diagnostic information.
- Teledentistry would be too expensive to set up.
- Teledentistry would save time compared with a referral letter.
- Teledentistry would necessitate an extra appointment for taking photographs.
- Teledentistry would increase surgery time spent with the patient.
- Teledentistry would reduce costs for the dental practices.
- Teledentistry would enhance clinical training and continuing education.

11. **On a scale of 1 (Strongly disagree) to 5 (Strongly agree) how would you agree with the following statements regarding dentists' perception of efficiency of teledentistry system:**
- Teledentistry would make referral of new patients more efficient.
- Teledentistry would improve communications between dentists (including dental therapists, GDP and consultants
- Teledentistry would enhance guidance and advice.
- Teledentistry would help shorten waiting lists.
- Teledentistry diagnosis is accurate via assessment of intra-oral images as in traditional clinical setting.

12. **On a scale of 1 (Strongly disagree) to 5 (Strongly agree) how would you agree with the following statements regarding dentists' perception of benefits of teledentistry system for patients**
- Teledentistry would be useful for patients in distant or rural locations.
- Teledentistry would be convenient for patients and well received by patients.
- Teledentistry would be helpful to monitor a patient's condition.
- Teledentistry would help reduce unnecessary travel to hospital.
- Teledentistry would help with patient information and education.
- Teledentistry would improve interaction and communication with patients.
- Teledentistry would save money for patients.

13. **On a scale of 1 (Not concerned at all) to 5 (Very concerned) how concern you are with the following statements regarding dentists' concern about practice-related use of information technologies:**
- Reliability of Equipments
- Technical incompatibility
- Patient confidentiality when images are sent online to the hospital.
- Gaining patient consent for referral via email
- Potential for tampering with computer images

THANK YOU!
